# Comparison of the safety and persistence of immunogenicity of bivalent HPV16/18 vaccine in healthy 9–14-year-old and 18–26-year-old Chinese females: A randomized, double-blind, non-inferiority clinical trial

**DOI:** 10.1016/j.vaccine.2023.10.041

**Published:** 2023-11-22

**Authors:** Juan Li, Li-Wei Shi, Ke Li, Li-Rong Huang, Jian-Biao Li, Yu-Lian Dong, Wei Li, Min Ji, Qing Yang, Ling-Yun Zhou, Lin Yuan, Xue-Mei Yan, Jing-Jing Chen, Zhi-Wei Jiang, Yang-Yang Qi, Rong-Cheng Li, Yan-Ping Li, Jie-Lai Xia, Bang-Wei Yu, Zhao-Jun Mo, Chang-Gui Li

**Affiliations:** a National Institute for Food and Drug Control, Beijing, China; b Guangxi Zhuang Autonomous Region Center for Disease Control and Prevention, Nanning, Guangxi, China; c Shanghai Zerun Biotechnology Co., Ltd., Shanghai, China; d Hezhou Center for Disease Control and Prevention, Hezhou, Guangxi, China; e Zhongshan Center for Disease Control and Prevention, Zhongshan, Guangxi, China; f Yuxi Zerun Biotechnology Co., Ltd., Yunnan, China; g Walvax Biotechnology Co. Ltd, Kunming, Yunnan, China; h Beijing Key Tech Statistical Technology Co., Ltd., Beijing, China; i Air Force Military Medical University, Xi’an, Shaanxi, China

**Keywords:** Human Papilloma Virus, Vaccine: girls, Immunogenicity, Safety, Tolerability

## Abstract

**Background:**

We assessed the safety, immunogenicity and antibody persistence of two- and three-dose schedules of the novel bivalent HPV16/18 vaccine (HPV-2, Walrinvax) in the per-protocol target population of initially seronegative 9–14 year-old girls, including a non-inferiority comparison with the three-dose schedule in 18–26 year-old women.

**Methods:**

This randomized phase 3b trial in Guangxi Zhuang Autonomous Region, China, involved healthy Chinese females in two age cohorts; 600 girls aged 9–14 years and 300 women aged 18–26 years. Girls were randomly assigned (1:1) to receive either two (Months 0,6) or three (Months 0,2,6) intramuscular doses of HPV-2. All participants were monitored for immunogenicity as neutralizing antibodies up to 36 months. Primary objectives were non-inferiority analyses of immunogenicity between two- and three-dose girl groups and adult women at Month 7; safety assessments were based on participant-completed diary cards.

**Results:**

All groups demonstrated marked increases in neutralizing antibodies against HPV 16 and 18 that persisted above baseline to 36 months. Month 7 responses in both girl groups were non-inferior to those in the women and were statistically higher after two-doses than girls or women who received three doses. GMTs waned after month 7, but then maintained a plateau level until month 36. Vaccination was well tolerated in all groups with no serious adverse events reported.

**Conclusions:**

Immune responses to two doses of HPV-2 vaccine in adolescent girls were non-inferior to those after three doses in young women, an age cohort in which clinical efficacy of HPV-2 against cervical cancer has been demonstrated.

## 1 Introduction

Cervical cancer due to infection by human papilloma virus (HPV) remains the third leading cause of cancer in Chinese women from 15 to 44 years of age [1]. Almost 110,000 Chinese women are diagnosed with cervical cancer annually and over 59,000 women die [2]. Despite the availability of effective HPV vaccines since 2006, their implementation in China only began in 2016/17. The high cost of these vaccines has been a barrier to their widespread use, emphasizing the need for more cost-effective alternatives [3].

In response to the need for a domestic HPV vaccine, Shanghai Zerun Biotechnology Co., Ltd. has developed Walrinvax (HPV-2), a recombinant Human Papillomavirus bivalent (Types 16, 18). This vaccine targets HPV strains 16 and 18, which are responsible for over 70% of HPV-related diseases. The vaccine has demonstrated safety and immunogenicity in females from 9 to 45 years in phase 1 and 2 studies [4], [5], and a phase 3 trial showed a protective efficacy of 78.6% (95% CI: 23.3–96.1) against CIN2+ lesions due to HPV 16 or 18 following a three-dose schedule of HPV-2 in 18–30-year-old Chinese women [6].

The target population for HPV immunization is girls under 15 years of age before they become sexually active and are exposed to HPV [7]. As part of the global strategy to eliminate cervical cancer the WHO has set a target to ensure 90% of girls are fully vaccinated with HPV vaccine by 15 years of age [7]. Further, analysis of recent studies has demonstrated that two-dose (0,6 months) and three-dose (0,2,6 months) HPV vaccine regimens induce comparable immunogenicity up to 5 years post-vaccination and are non-inferior to responses in women that have been associated with protective efficacy against cervical precancerous lesions and cervical cancer [8], [9]. This has resulted in the 0,6 month two-dose schedule being recommended by the WHO [10] and such schedules are now approved for girls aged 9–14 years by the US CDC [11] and in Europe by the European Medicines Agency [12].

The present study was performed to demonstrate non-inferiority of the neutralizing antibody responses against HPV 16 and 18 following two doses (0,6 months schedule) or three doses (0,2,6 months) of Walrinvax vaccine compared with the responses to the three-dose regimen in adult women (18–26 years old). We also assessed the safety and reactogenicity of the vaccine, and the persistence of these immune responses up to three years after receipt of the first dose.

## 2 Methods

This Phase 3b study was conducted at two clinical study sites in Hezhou City and Zhongshan County of the Guangxi Zhuang Autonomous Region from February 2016 to July 2019. The protocol was approved by the independent ethics committee Guangxi Provincial Centre for Disease Control and Prevention and registered on ClinicalTrials.gov, identifier NCT02740777. The study was performed in compliance with International Council for Harmonisation (ICH) and Good Clinical Practice (GCP) guidelines. Written informed consent was obtained from all participants and the parents/guardians of the adolescent girl cohort before enrollment. The primary objective was to demonstrate non-inferiority of the immune response to two doses of HPV-2 in adolescent girls compared with the response to three doses in young women. Secondary objectives included a non-inferiority comparison of immune responses to three doses in adolescent girls vs. young women, and safety and reactogenicity of HPV-2. The study was extended to assess the persistence of the immune responses to 3 years following the first vaccination, 30 months after the last vaccination.

Eligible adolescent girl participants were aged between 9 and 14 years at the time of enrollment and healthy as established by their medical history and clinical examination. Eligible women participants were aged between 18 and 26 years, free of obvious health problems as established by their medical history and clinical examination, were not pregnant (confirmed by urine pregnancy test) nor planning to become pregnant until at least Month 7 of the study, and those who were sexually active agreed to use effective contraception until then. Main exclusion criteria for both cohorts included previous HPV vaccinations, known allergy to any vaccine component, prior or ongoing immunosuppression, and any acute illness within the seven days preceding enrollment or any fever (axillary temperature >37.0 °C) at the time of vaccination.

### 2.1 Vaccine

The study vaccine, Walrinvax (HPV-2), is a recombinant bivalent HPV (types 16 and 18) vaccine developed by Shanghai Zerun Biotech Co., Ltd, China, and produced using recombinant DNA technology to produce HPV 16 and 18 L1 proteins using the yeast *Pichia pastoris*. Proteins are purified and self-assemble into virus-like particles (VLP) which can be absorbed on aluminum phosphate for vaccine formulation. The vaccine contained 40 μg of HPV16 and 20 μg of HPV18 L1 VLPs protein adsorbed to 225 μg of aluminum phosphate suspended in 0.5 ml of buffered saline (0.32 M sodium chloride, 10 mM L-histamine, 0.025 mg Polysorbate 80). The vaccine batch used (Lot A201501202) was prepared in a Good Manufacturing Practices compliant facility and verified by the National Institutes for Food and Drug Control before use. Vaccine was administered by intramuscular injection in the lateral deltoid muscle of upper arm.

### 2.2 Procedures

Adolescent girl participants were randomly allocated 1:1 to two groups to receive either two doses (at Months 0 and 6) or three doses (at Months 0, 2 and 6) of Walrinvax, while young women all received three doses at Months 0, 2 and 6. On Day 0, following enrollment and after a baseline blood draw (and a negative urine pregnancy test in young women) the first dose was administered. In the three-dose groups a second dose was administered at Month 2 and all participants received their second or third dose at Month 6. Participants were monitored for 30 min after each vaccination for any immediate reactions. They were supplied with study diary cards on which to record solicited local reactions (pain, induration, redness, swelling, pruritis at the injection site) and systemic adverse events (fever, allergy, headache, fatigue, nausea/vomiting, diarrhea, myalgia) for 7 days which were returned on Day 8. Any unsolicited adverse events were recorded from enrollment to Month 7, and any serious adverse events (SAE) were recorded from enrollment to Month 12, regardless of cause, and participants or parents/legal guardians were asked to contact the investigator immediately in the event of an SAE. An SAE was defined as any untoward medical occurrence that resulted in death, was life-threatening, resulted in persistent or significant disability/incapacity, necessitated hospitalization or prolongation of existing hospitalization, or was a congenital anomaly/birth defect in the offspring of a study participant.

### 2.3 Immunogenicity

Sera prepared immediately from blood samples obtained from all participants before administration of vaccine at baseline and then at follow-up visits at Months 7, 12, 24 and 36 were used to test for neutralizing antibodies against HPV16 and HPV 18 in the three study groups. Sera were also obtained from a subset of 100 participants in each group at Month 6 for an exploratory assessment of the response after one and two doses. Immunogenicity testing was performed at the China National Institute for Food and Drug Control using a highly sensitive, automated, high-throughput, pseudovirion-based neutralization assay (PNBA) specific for neutralizing epitopes in the HPV-L1 proteins of HPV16 and 18 as previously described [4], [5]. Briefly, 293 FT cells were placed in 96-well cell culture plates at 15,000 cells/well with 100 μL of growth medium and incubated for 16–24 h. The pseudovirions were diluted to ∼15,000 TCID_50_/mL, and 60 μL of diluted pseudovirions were mixed with 60 μL of serially diluted sera. The mixtures were incubated at 25 °C for 1 h and then transferred into plates pre-seeded with 293 FT cells at 100 μL/well and incubated for 68–76 h following which expression of green fluorescent protein (GFP) was observed by fluorescence microscopy. Neutralization titers are expressed as the reciprocal of the dilution causing a 50% reduction in GFP expression compared with a negative control and a titer ≥40 was considered to indicate seropositivity for HPV16 and 18, respectively.

### 2.4 Statistics

Non-inferiority was assessed based on neutralizing antibodies against HPV 16 and 18. The endpoints to determine the primary objective were the neutralizing antibody responses against HPV 16 and 18 measured at Month 7, one month after the last of two or three doses of HPV-2. Non-inferiority comparisons for each HPV type were calculated between the geometric mean titers (GMTs) in the two groups of 9–14-year-old girls who received either two or three doses of HPV-2 compared with the GMT in 18–26-year-old women one month after a third vaccination. Non-inferiority between two groups was demonstrated if the lower limit of the 95% confidence interval (CI) of the GMT ratio of the two groups was greater than 0.5 (the 95% CI lower limit of the inter-group difference after 10-based logarithmic conversion should be greater than −0.3). The primary objective was calculated in the per protocol set (PPS) consisting of those who were seronegative to the appropriate HPV type at Day 0, had received all their assigned vaccinations punctually according to schedule, provided paired sera samples with valid serology results from Months 0 and 7, and did not have any protocol violations that could interfere with the participant’s immune response. With at least 235 participants in each group, the study had greater than 90% power to establish both immunogenicity hypotheses at a 1-sided significance level of 0.025. Assuming a 15% withdrawal rate and some participants being seropositive at baseline, we determined that a sample size of 300 participants per group, a total of 900 participants, was required. Statistical analyses were performed using PASS11 software.

## 3 Results

**Fig. 1:**
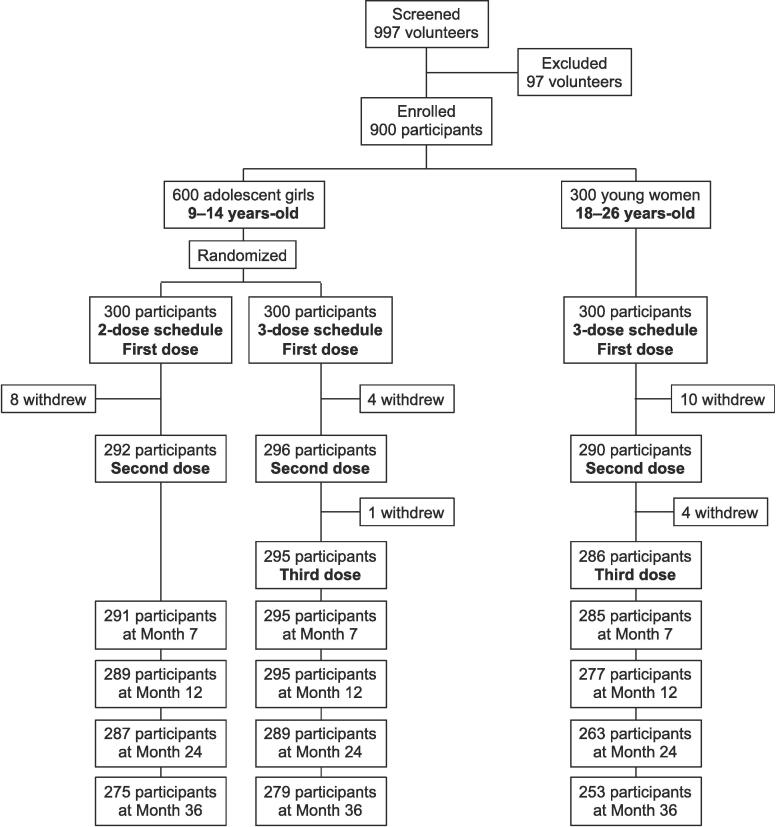
Study flow chart.

A total of 997 female volunteers were screened for inclusion in the study and 97 were excluded before 900 eligible participants were enrolled during February and March 2016; 600 girls aged 9 to 14 years randomly allocated to two equal groups and 300 women aged 18 to 26 years (Fig. 1). A total of 843 (93.7%) and 852 (94.7%) participants were available at Month 7 for the Per Protocol primary objective analysis of HPV 16 and HPV 18, respectively; 796 (88.4%) presented for the persistence analysis at Month 36. The demographics in the two adolescent girl groups were similar with median ages of 11.6 and 11.5 years, compared with 24.1 years in the young women group (Table 1). Most adolescent girls were seronegative for either HPV 16 or 18, with 4 seropositive for HPV 16 and two for HPV 18. Among the young women 11 participants were seropositive for HPV 16, 3 for HPV 18 and 2 for both HPV 16 and 18.

**Table 1:** Demographic characteristics of the participants in three study groups.

		**Girls (9–14 years)**		**Women (18–26 years)**
**Characteristic**		**Two doses**	**Three doses**	**Three doses**
	**N =**	300	300	300
**Age, (yr)**	Median (min, max)	11.6 (9.0, 15.0)	11.5 (9.0, 15.0)	24.1 (18.1, 26.9)
**Height, (cm)**	Median (min, max)	145 (119, 169)	145 (115, 164)	156 (140, 170)
**Weight, (kg)**	Median (min, max)	35.8 (20.0, 66.0)	36.0 (19.0, 70.0)	49.0 (35.5, 95.0)
**Body Mass Index, (kg/m^2^)**	Median (min, max)	17.3 (12.6, 31.0)	17.5 (11.7, 30.6)	20.5 (15.4, 34.1)
**Ethnic group, n (%)**				
Han		254 (84.7)	246 (82.0)	257 (85.7)
Zhuang		18 (6.0)	22 (7.3)	14 (4.7)
Yao		28 (9.3)	32 (10.7)	29 (9.7)
**Baseline seropositivity rates,** n (%)				
Seronegative		296 (98.3)	298 (99.3)	284 (94.7)
HPV 16		2 (0.7)	2 (0.7)	11 (3.7)
HPV 18		2 (0.7)	0	3 (1.0)
HPV 16 & 18		0	0	2 (0.7)

### 3.1 Immunogenicity assessments

**Fig. 2:**
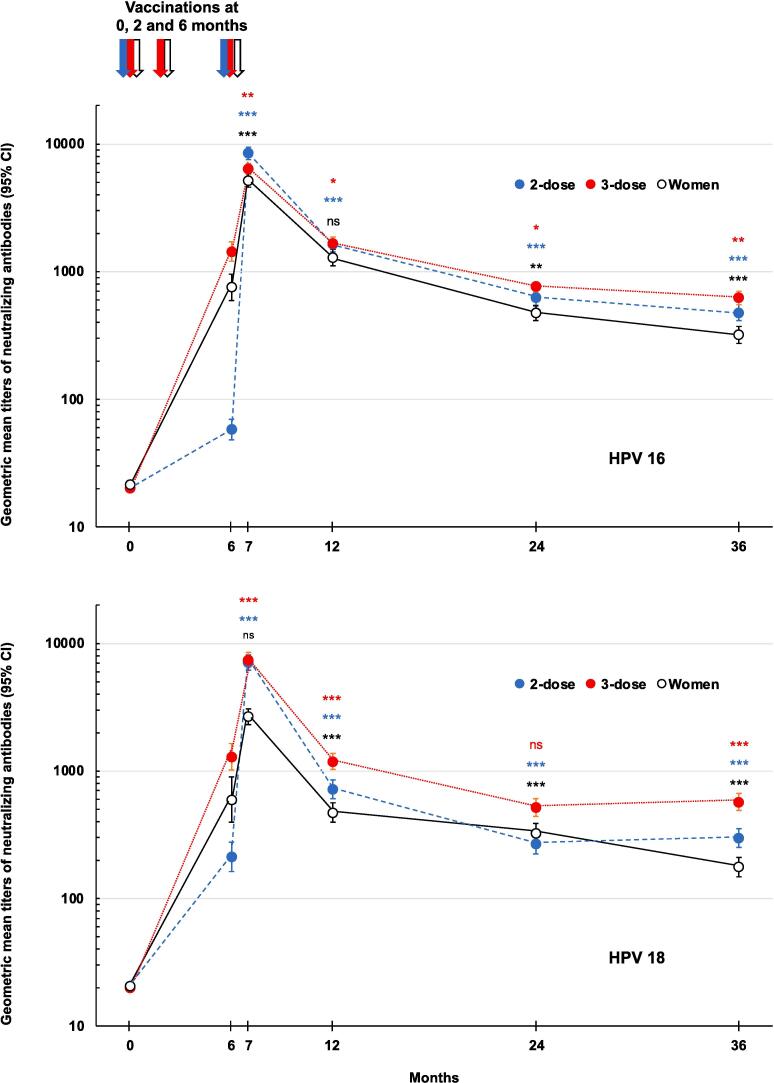
Kinetics of anti-HPV 16 (upper panel) and HPV 18 (lower panel) immune response in the three study groups (PPS). Values from 247 to 300 participants at each timepoint, except subsets of each group (n = 95, 95 & 88) at Month 6. Statistical comparisons between groups are shown as * p < 0.05; ** p < 0.01 and *** p < 0.001 or non-significant (ns) for comparisons of 2-dose girls vs. 3-dose women (red), 3-dose girls vs. 3-dose women (blue) and 2-dose vs. 3-dose girls (black). (For interpretation of the references to colour in this figure legend, the reader is referred to the web version of this article.)

Then immune responses against HPV 16 and 18 at each study timepoint are illustrated in Fig. 2 for the 36 months of the study. All three groups displayed an initial rapid increase in GMTs followed by a biphasic decline, a rapid decline to Month 12 followed by a slower rate to Month 36. At Month 6 anti-HPV 16 GMTs were higher in both adolescent girls (1437 [95% CI: 1203–1715]) and young women (755 [593–961]) after two doses, than the adolescent girl group (58.0 [48.2–69.9]) which had only received one dose at Month 0. Similar observations were made for anti-HPV 18 titers which were 1289 (1015–1637) in adolescent girls and 596 (396–897) in young women after two doses, and 213 (164–277]) after one dose in adolescent girls. However, by Month 7, one month after receiving the second or third doses at Month 6, the two adolescent girl groups had similar GMTs for both anti-HPV 16 and 18 antibodies, which appear to be higher than the young women group. This was confirmed by the analysis for the primary outcome that showed the statistical non-inferiority of these responses in both adolescent girl groups compared with the young women (Table 2). Respective GMTs for anti-HPV 16 antibodies were 8511 (95% CI: 7586–9550) and 6457 (5754–7079) in two- and three-dose adolescent girl groups, compared with 5129 (4677–5754) in the young women. GMT ratios for antibodies against HPV 16 were 1.62 (95% CI: 1.41–1.91) and 1.23 (1.07–1.45) for two- and three-dose adolescent girls groups compared with the young women group, respectively. In both cases the lower 95% CI was higher than 0.5 and so met the non-inferiority criterion. Similarly, Month 7 GMTs of antibodies against HPV 18 were 7079 (6310–8128) and 7413 (6457–8511) in two-dose and three-dose adolescent girl groups compared with 2692 (2344–3090) in the young women group with GMT ratios of 2.69 (95% CI: 2.19–3.24) and 2.82 (2.29–3.39), respectively. As with HPV 16, the lower 95% CI was higher than 0.5 confirming that responses in both adolescent girl groups met the non-inferiority criterion.

**Table 2:** Non-inferiority assessments of geometric mean antibody titers (GMT) against HPV 16 and 18 one month at Month 7, after last vaccination in two- and three-dose girls groups and three-dose women group.

	**Girls** **(9–14 years)**		**Women** **(18–26 years)**	**GMT ratios of girls vs. women** (95% CI)		**GMT ratios of 2-dose vs. 3-dose girls** (95% CI)
**HPV vaccination**	**Two doses**	**Three doses**	**Three doses**			
**N =**	288	288	267			
**Anti-HPV 16** **GMT** (95% CI)	**8511** (7586–9550)	**6457** (5754–7079)	**5129** (4677–5754)	**2-dose**	**1.62** (1.41–1.91)	**1.32** (1.15–1.55)
				**3-dose**	**1.23** (1.07–1.45)	
**N =**	288	290	274			
**Anti-HPV 18** **GMT** (95% CI)	**7079** (6310–8128)	**7413** (6457–8511)	**2692** (2344–3090)	**2-dose**	**2.69** (2.19–3.24)	**0.95** (0.79–1.15)
				**3-dose**	**2.82** (2.29–3.39)	

**Fig. 3:**
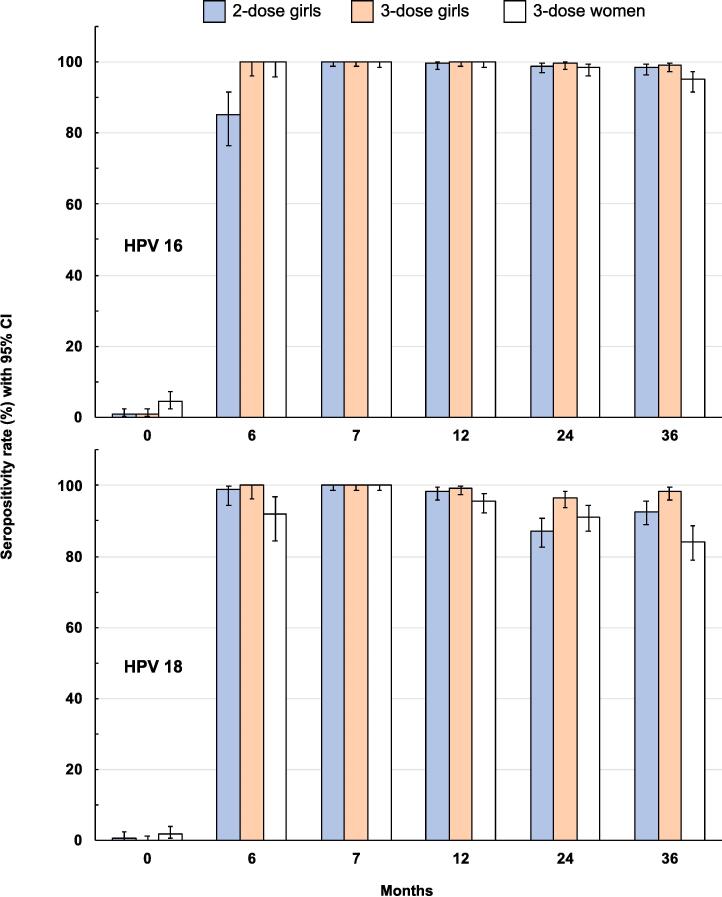
Persistence of anti-HPV seropositivity* in 2- and 3-dose girl groups and the 3-dose women group - HPV 16 in upper panel, HPV 18 in lower panel. * Seropositivity defined as a titer ≥ 40.

There were no differences in seropositivity rates (Fig. 3) at Month 7, once the two- and three-dose regimens had been completed, when all three study groups achieved 100% showing that all initially seronegative vaccinees, irrespective of age or vaccination regimen, had seroconverted against both antigens.

### 3.2 Antibody persistence

Antibody GMTs against HPV 16 were consistently comparable or significantly higher in the two- and three-dose adolescent girl groups than the three-dose women group up to Month 36 (Fig. 2). Throughout this period, GMTs against HPV 18 in the two- and three-dose adolescent girl groups were consistently higher or comparable to those in the three-dose women group. From a peak at Month 7, one month after the last dose, there was a general decline until a plateau was reached at around Month 24 to 36. The GMT ratios between the two-dose and three-dose adolescent girl groups and the three-dose women group for both HPV types ranged from 1.5 to 3.24 at Month 36. The lower bound of the 95% CI for the GMT ratios remained above 0.5 for both HPV types, indicating persistence of the noninferiority observed at Month 7 through Month 36.

Waning antibody titers had little effect on the seropositivity rates (Fig. 3). By Month 36, 30 months after the last vaccination, 98.5% and 99.3% of the two- and three-dose girl groups remained seropositive against HPV 16, compared with 95.1% of the women group. Seropositivity rates against HPV 18 were 92.7% and 98.2% in the two- and three-dose girl groups at Month 36, but the rate had fallen to 84.2% in the young women.

### 3.3 Safety and tolerability

Vaccination with HPV-2 was generally well tolerated with no safety signals. There were no deaths during the study and of the five serious adverse events (SAE) reported, one case of appendicitis in a young woman and cases of bronchitis, skin infection, tonsillitis and a burn in three girls in the three-dose group), none was considered to be related to vaccination. Unsurprisingly, more girls in the three-dose group reported having an adverse event than girls in the two-dose group, presumably due to the greater number of injections, and adolescent girls reported more AEs than young women (Table 3). Most solicited local reactions were local injection pain, reported at similar rates in all three groups (17.7–20.7%), and were all described a mild or moderate in severity. Other local reactions were reported less frequently (<5%) and at similar rates across the three groups.

**Table 3:** Summary of adverse events (AE) reported by the participants in the three study groups.

	**Girls** **(9–14 years)**		**Women** **(18–26 years)**
**Participants reporting adverse events,** n (**%**)	**Two doses**	**Three doses**	**Three doses**
**N =**	300	300	300
**At least one AE**	158 (**52.7**)	190 (**63.3**)	134 (**44.7**)
**Local reaction (any)**	55 (**18.3**)	66 (**22.0**)	53 (**17.7**)
Pain	53 (**17.7**)	62 (**20.7**)	53 (**17.7**)
Itching	8 (**2.7**)	13 (**4.3**)	7 (**2.3**)
Redness	5 (**1.7**)	3 (**1.0**)	2 (**0.7**)
Swelling	2 (**0.7**)	3 (**1.0**)	1 (**0.3**)
Induration	0	0	1 (**0.3**)
**Systemic adverse event (any)**	108 (**36.0**)	126 (**42.0**)	93 (**31.0**)
Fever	88 (**29.3**)	107 (**35.7**)	75 (**25.0**)
Headache	19 (**6.3**)	21 (**7.0**)	17 (**5.7**)
Fatigue	11 (**3.7**)	13 (**4.3**)	11 (**3.7**)
Diarrhea	6 (**2.0**)	10 (**3.3**)	7 (**2.3**)
Nausea/vomiting	6 (**2.0**)	7 (**2.3**)	8 (**2.7**)
Myalgia	3 (**1.0**)	3 (**1.0**)	4 (**1.3**)
Allergic reaction	3 (**1.0**)	4 (**1.3**)	4 (**1.3**)
**Unsolicited adverse event**	74 (**24.7**)	101 (**33.7**)	65 (**21.7**)
**Serious adverse event (SAE)**	0	3 (**1.0**)	1 (**0.3**)
**Discontinued due to AE**	0	0	1 (**0.3**)*
**Pregnancies – total**	Not applicable		6
Baby born			4 ^#^
Induced abortion			2

* One woman withdrew with injection site pain, fever, headache, fatigue and myalgia after the first vaccination.

^#^ 3 normal vaginal deliveries, 1 caesarean section, all infants normal and healthy.

The most frequently reported solicited systemic adverse event was fever, an axillary temperature >37 °C, but there were only six reports of fever >39 °C which occurred in 2 and four girls in the two- and three-dose groups, respectively. Headache was the only other systemic AE reported by more than 5% of any the study groups. All systemic AEs resolved without sequelae. Unsolicited AEs, reported by 21.7% of young women and 24.7% and 33.7% of the two- and three-dose adolescent girl groups, mainly consisted of upper respiratory tract viral infections, upper respiratory tract infections, cough, fever, pharyngitis and abdominal pain. A total of 13 unsolicited AEs were graded as severe, but none of these severe cases were considered to be related to vaccination by the investigators.

Despite the contraception requirement there were six pregnancies in the cohort of young women (Table 3) which resulted in two voluntary induced abortions and four births, three by vaginal delivery and one caesarean. All babies were healthy at delivery.

## 4 Discussion

The primary objectives of this study were successfully met as we demonstrated that in girls aged 9 to 14 years immune responses to HPV 16 and HPV 18 after two or three doses of the novel HPV-2 vaccine, Walrinvax, were non-inferior to those observed in young women aged 18 to 26 years after three doses. Neutralizing antibody responses against both HPV 16 and HPV 18 in adolescent girl groups who received either a two- or three-dose HPV-2 regimen were not only non-inferior but were actually higher than those observed in the young women one month after the last vaccination of their three-dose regimen. The robust immune response in the young girls was maintained for at least 30 months post-vaccination after either two or three doses with non-inferiority of the response in girls compared with young women persisting for the 30 months of follow-up. Furthermore, all vaccinations in either two- or three-dose schedules, were well tolerated by the girls with no vaccine-related serious adverse events reported, and generally mild to moderate reactogenicity mainly consisting of injection site pain and low-grade fever.

Our immunogenicity observations are important for several reasons. Firstly, they bridge the immune response to HPV-2 in the target population of adolescent girls to the response in an older cohort of young women in which 78.6% efficacy of HPV-2 vaccine against cervical precancer associated with HPV 16 or 18 has been demonstrated [6]. They also demonstrate that HPV-2 can be used in a two-dose schedule in adolescent girls which has been proven to be effective with other HPV vaccines [8], [9]. All currently approved bivalent, quadrivalent, and nonavalent HPV vaccines demonstrate similar immunogenicity, efficacy, and effectiveness in preventing cervical precancer lesions primarily induced by HPV types 16 and 18 [13]. These data have led to two-dose regimens now being recommended by the WHO [10] and approved in the United States [11] and Europe [12]. Our study findings align with the WHO recommendations. The potential for a two-dose regimen will further enhance the accessibility to Walrinvax as fewer doses will not only mean lower administration costs, but also a lower logistical burden on healthcare systems and individuals, particularly in areas where multiple visits to healthcare facilities may be challenging. This study, therefore, supports the two-dose schedule as a potential strategy to both improve compliance and to make available more doses for more vaccinees to provide more equitable vaccination coverage and prevent HPV-related diseases.

Our results are consistent with previous reports. A multi-center study in Canada, Germany, Italy, Taiwan, and Thailand found non-inferiority of a two-dose regimen of the ASO4-adjuvanted bivalent HPV vaccine in 9–14-year-old girls compared with three doses in 15–25-year-olds [13]. In Canada, antibody responses to two doses of the quadrivalent HPV vaccine in girls aged 9 to 13 years were shown to be comparable with those in women aged 16 to 26 years who received three doses [14]. Finally, responses to two doses of the nonavalent vaccine in 9–14-year-old girls or boys were non-inferior to three doses of the same vaccine in young women in a multinational study [15]. In China, another group studied a bivalent HPV vaccine produced in *E. coli* in 9–14-year-old girls versus 18–26-year-old women [16]. They demonstrated that responses measured as IgG and neutralizing antibodies one month after the second of two doses in the girls were non-inferior to those one month after the third dose in the young women. In another study of the same vaccine the authors found over 99% of 9–14-year-old Chinese girls were still seropositive against both HPV 16 and 18 two years after the second dose, when titers of IgG antibodies measured by ELISA were comparable with those observed in 18–26-year-olds after three doses [17].

More recent investigations have focused on the potential of a one-dose HPV vaccination regimen including a large study in 15–20 year-old African women which has demonstrated similar effectiveness of one or two dose HPV regimens with either the bivalent Cervarix or nonavalent Gardasil-9 vaccines [18]. High seropositivity was found in a second study in 9–14-year-old girls two years after a single dose of either of the two vaccines [19]. These studies, together with a systematic review of all data on one-dose HPV vaccination [20], lead the WHO SAGE to recommend off-label use of a single-dose schedule for routine immunization of the target 9–14 years age group to make vaccination programs more efficient and affordable [21]. At present, we do not have data on one-dose immunogenicity and efficacy of Walrinvax. Further research into a potential one-dose schedule for Walrinvax could help maximize population coverage and facilitate widespread vaccine implementation, an area of important future study.

There are limitations in our trial which must be acknowledged. Firstly, this study bridges immunogenicity data between cohorts of adolescent girls and young women, and the immunogenicity follow-up in the present was stopped at 36 months, 30 months after the vaccination. Clinical efficacy of a three-dose regimen of Walrinvax has been studied in a cohort of women aged 18–30 years with only 48 months follow-up [6]. In the absence of a serologic correlate of protection we are assuming that equivalent immunogenicity will lead to equivalent clinical efficacy when administered to younger girls, which remains to be proven, but the necessary longer follow-up assessments to demonstrate the durability of this immune response up 11 years after vaccination are currently underway. Although these studies were performed in Chinese populations only there is currently no evidence of differences in vaccine efficacy or immune response among different geographic areas and the robust immune response observed in the target population of girls aged 9 to 14 years suggests that the vaccine is likely to be effective in other populations as well. However, as we are seeking WHO prequalification for Walrinvax to provide supply for global use, further research is needed to evaluate its effectiveness and safety in different geographical and demographic settings. Due to the limited access to WHO-prequalified HPV vaccines in China at the time of our study we were unable to use such a vaccine as control. Similarly, the fact that we did not have access to international anti-HPV type 16/18 sera standards to correlate our immunogenicity data with other studies. However, this does not affect the main objective of bridging responses in young girls to adult women using the same vaccine which is the basis for WHO approval of the vaccine in a two-dose schedule [21].

Our present data from this bridging study indicate that Walrinvax meets the necessary criteria for licensure according to the WHO guidelines as it shows at least equivalent immunogenicity in the target population of 9–14-year-old girls to an adult cohort in which clinical efficacy has been demonstrated. Availability of another HPV vaccine will help overcome supply and cost issues which are currently preventing China achieving the goals of HPV immunization.

## 5 Conclusions

In conclusion, this study provides important evidence supporting the use of a two-dose regimen of Walrinvax in 9–14-year-old girls. The findings contribute to the growing body of evidence on the effectiveness and safety of two-dose HPV vaccine regimens and highlight the potential of implementation of a two-dose Walrinvax vaccination schedule to realize the economic and health benefits this bivalent vaccine could provide in the global effort to prevent HPV-related diseases.

## Declaration of Competing Interest

The authors declare the following financial interests/personal relationships which may be considered as potential competing interests: B-W Yu, K Li, M Ji, Q Yang and L-Y Zhou are full-time employees of the manufacturer. Other authors declare no conflicts of interest.

## Data Availability

Data will be made available on request.
